# Spirituality/Religiosity as a Therapeutic Resource in Clinical Practice: Conception of Undergraduate Medical Students of the Paulista School of Medicine (*Escola Paulista de Medicina)* - Federal University of São Paulo (*Universidade Federal de São Paulo*)

**DOI:** 10.3389/fpsyg.2021.787340

**Published:** 2021-12-24

**Authors:** Silvia Borragini-Abuchaim, Luis Garcia Alonso, Rita Lino Tarcia

**Affiliations:** ^1^Center for the Development of Higher Education in Health (CEDESS), Paulista School of Medicine, Federal University of São Paulo, São Paulo, Brazil; ^2^Department of Morphology and Genetics, Paulista School of Medicine, Federal University of São Paulo, São Paulo, Brazil

**Keywords:** spirituality, religiosity, medical education, clinical practice, undergraduate medical students

## Abstract

**Introduction:** The high degree of religious/spiritual involvement that brings meaning and purpose to a patients’ life, especially when they are weakened by pain, is among the various reasons to consider the spiritual dimension in clinical practice. This involvement may influence medical decisions and, therefore, should be identified in the medical history of a patient (anamnesis).

**Objective:** To verify the opinion of undergraduate medical students of the Paulista School of Medicine – Federal University of São Paulo regarding the use of a patient’s Spirituality/Religiosity as a therapeutic resource in clinical practice.

**Method:** Quantitative approach of the transversal analytical observational type. The sample was composed of academics’ medical program, from the first to the sixth year, regularly enrolled in 2017. Data collection was performed with a standardized questionnaire divided into three sections: sociodemographic profile; Duke University Religious Index; Spirituality/Religiosity in the clinical and academic context.

**Results:** Participated in the survey 72% of the enrolled students, of which 61.4% had religious affiliation, 26.2% declared themselves agnostic and 12.4% atheists. All of them proposed to answer questions about the insertion of Spirituality/Religiosity in the patient care process. Through the Duke Religiosity Index, we evaluated the importance of religiosity in the student’s personal life and the pertinence of religiosity as a therapeutic insertion for medical treatment. Regarding the clinical and academic context, most participants considered relevant the proposition of didactic-pedagogical actions in medical education related to the spiritual dimension of the patient.

**Conclusion:** We conclude, through our research, that the insertion of the Spirituality/Religiosity of the patient as a therapeutic resource in clinical practice is feasible for most undergraduate students in Medicine of the Escola Paulista de Medicina – Universidade Federal de São Paulo (Paulista School of Medicine - Federal University of São Paulo). The result of the research, although it shows only the opinion of medical students at a Brazilian university, indicates that Spirituality/Religiosity is already part of the contemporary medical universe.

## Introduction

This study involves the “triad”: Religiosity, Spirituality, and Health. To better understand the concept of Religiosity, we will define Religion as an organized system of beliefs, practices, rituals, and symbols designated to facilitate access to the sacred and the transcendent (God, Greater Force, and Supreme Truth.). Religiosity, thus, corresponds to how much an individual believes, follows, and practices a religion (Koenig et al., 2001).

The first challenge was to seek a consensual definition of Spirituality in the scientific literature of health. There are several definitions, some totally dissociated from the meaning of Religiosity, and others that are intertwined with it. According to Koenig et al. (2001), Spirituality can be conceptualized as a personal quest to understand issues related to the purpose of life, its meaning, as well as relations with the sacred or transcendent that may or may not lead to the development of religious practices or formation of religious communities. Koenig (2012b) does not conceive a definition of Spirituality that is totally distanced from the “sacred or transcendent,” but states that, because Spirituality is an aspect of human experience, the use of a broader definition, such as those presented by Association of American Medical Colleges [AAMC] (1998), Anandarajah and Hight (2001), and Puchalski et al. (2009) makes sense in clinical practice.

Puchalski et al. (2009) evidence this scope when they define Spirituality as an aspect of humanity that deals with the way individuals seek and express meaning and purpose, as well as with the way in which they express their connection with the moment, with oneself, with others, with nature, and with the sacred. According to Avezum and Esporcatte (2019), regardless of their belief, every human being has at least one form of Spirituality based on existential philosophy, finding meaning, purpose and fulfillment in life.

Medicine is not a reference area for the academic study of religions but provides intertextuality and interdisciplinarity with the Study of Religions, Anthropology and Sociology.

According to Crawford (2005), for scientists of religion, there is no universally accepted definition of religion due to the wide variety of existing theories. The English philosopher of religion and theologian, John Hick, states that religion has a different conception for the various areas of knowledge, such as anthropology, sociology, psychology, philosophy, and for different religious traditions. We conclude that, for scientists of religion, not only Spirituality, but also Religion and Religiosity do not have a definition of consensus.

Anthropologist Clifford Geertz defines religion as a system of symbols that acts to “establish powerful, penetrating and lasting dispositions and motivations in men by formulating concepts of a general order of existence and wearing these conceptions with such an aura of factually that the dispositions and motivations seem singularly realistic” (Geertz, 1989, p. 67).

Émile Durkheim, a 19th-century French psychologist, philosopher, and sociologist, analyzes religion from the perspective of a *collective consciousness.* It presents in its functionalist theory, religion as a cultural/social subsystem, which has a conciliatory and unifying function in moments of existential crises that are lodged within society (Durkheim, 2014).

Regarding the concept of Health, the World Health Organization (WHO) presents the following definition: “Health is a state of complete physical, mental and social well-being and not merely the absence of disease or infirmity” (World Health Organization [WHO], 1946). The biomedical paradigm gave way to the biopsychosocial paradigm. Historically, Science and Religion have always walked together, from antiquity to the Renaissance. The rupture was solidified with the advent of the Enlightenment and the French Revolution (Moreira-Almeida, 2009; Numbers, 2009a,b). A tentative rapprochement between religion and medicine took place in 1910, with the publication of an article by Johns Hopkins University Professor of Medicine, Sir William Osler, with reflections on the healing powers of faith, where he advised that a clinician needs to be attentive to faith, this powerful force present in patients (Osler, 1910). In 1984, a movement of representatives from WHO member countries began to discuss and propose the inclusion of spiritual well-being in the concept of Health. However, the difficulty in finding a consensus in the conceptualization of Spirituality, due to cultural differences and religious traditions, impaired the discussion and led to the non-approval of the proposal (World Health Organization [WHO], 1999). Although spiritual well-being is not included in the definition of Health^1^, in 2002, in a cross-cultural perspective, the WHO developed the SRPB Module *(Spirituality, Religion and Personal Beliefs)* for its Quality-of-Life Instrument to add the spiritual dimension as a domain. The WHOQOL-SRPB field test instrument has 32 questions, covering aspects of quality of life related to Spirituality, Religiosity, and Personal Beliefs (SRPB) and should be used in conjunction with the WHOQOL-100 (WHOQOL-SRPB, 2002). The Portuguese version and validation were produced by Panzini et al. (2011).

Because we recognize that the approach of a patient’s Spirituality/Religiosity is extremely important in clinical practice and, at the same time, totally relegated, we decided to focus on this theme. We carried out research to identify the opinion of undergraduate medical students on the approach of a patient’s spiritual dimension as a therapeutic resource in clinical practice. We also wanted to know if they were in favor of the proposal of didactic-pedagogical actions related to a patient’s spiritual dimension in medical education (Borragini-Abuchaim, 2018).

We investigated the students’ religious affiliation and Religiosity to know how much this aspect would influence their perception of the therapeutic use of a patient’s Spirituality/Religiosity. We chose to apply the Duke University Religious Index (DUREL) (Koenig et al., 1997), a short and simple scale that provides relevant data (King and Koenig, 2009). The DUREL version in Portuguese was developed by Moreira-Almeida et al. (2008), and the validation was done by Lucchetti et al. (2012a) and Taunay et al. (2012), the latter verified that DUREL was reliable and valid for use in university populations.

Regarding the clinical context of the approach to the spiritual dimension of the patient, we verified in the literature that obtaining the spiritual history (spiritual anamnesis) does not happen effectively in the medical conduct^2^. It could be included, naturally, in the patient’s medical history (anamnesis), shortly after the social/family history, preceding the clinical examination. It is important to emphasize that spiritual anamnesis should not be coercive (Koenig, 2012a)^3^. There is scientific evidence on the therapeutic efficacy of Spirituality/Religiosity on physical and mental health and quality of life, beneficial effects on a patient’s adhering to treatment and positive interference in prognosis, improved doctor-patient relationship, and influence in medical decisions (Chibnall and Brooks, 2001; Peres et al., 2007; Lucchetti et al., 2010; Koenig, 2012a,b, 2015). Several studies have proven patients’ predisposition to share their beliefs with their physician (Anandarajah and Hight, 2001; Puchalski, 2006; Koenig, 2012a,b; Saad et al., 2015). The simple taking of spiritual history makes the patient feel comfortable to make use of his religious/spiritual beliefs as an adjunct to the medical treatment (Koenig, 2012a,b). Moreover, religious/spiritual involvement brings meaning and purpose to the lives of most patients, especially when they are weakened by pain (Dal-Farra and Geremia, 2010; Moreira-Almeida et al., 2010; Koenig, 2012a,b; Lucchetti et al., 2012b). Pargament et al. (2001) report that some misinterpreted religious traditions can provoke *negative coping*, which happens on average with 15% of patients, who believe that God has stopped loving them, that they are being punished, that they have been abandoned, and feel deeply frightened. The authors state that the spiritual history will allow a physician to identify these patients’ spiritual need and request the presence of a religious leader who can comfort them and get them out of this distressing situation. The broad search we carried out in the main databases on the subject generated a publication of our findings regarding the historical trajectory and the current state of the art from the perspective of Spirituality/Religiosity as therapy in patient care (Borragini-Abuchaim et al., 2019).

There is strong support in the literature suggesting that, with appropriate training in Spirituality/Religiosity, physicians are likely to perform spiritual anamnesis as a routine practice, since most of them are favorable to the approach. Training would also support the overcoming of obstacles that prevent the inclusion of the spiritual dimension in clinical practice, such as lack of time, lack of training or experience in taking a spiritual history, discomfort and insecurity in addressing the subject due to lack of knowledge, and fear of imposing one’s own beliefs on the patient (Anandarajah and Hight, 2001; Monroe et al., 2003; Puchalski, 2006; Peres et al., 2007; Dal-Farra and Geremia, 2010; Lucchetti et al., 2010, 2012b; Koenig, 2012a,b; Saad and De Medeiros, 2012; Reginato et al., 2016; Puchalski et al., 2020). Puchalski et al. (2009) also draw attention to the benefit it would bring to physicians themselves since they would access their own Spirituality during the process. In 2020, we published these and other bibliographic findings related to the academic context of Spirituality/Religiosity (Borragini-Abuchaim et al., 2020).

Globalization and over-rationalism lead 21st century man to automatism and dehumanization, directing the being to melancholy and disenchantment with life itself. Spirituality regardless of any religious belief, when inserted in the medical curriculum can be used as another therapeutic resource in clinical practice. The purpose should not be the discussion of religious beliefs, but rather the instrumentalization of the student for conscious and efficient interventions in the approach and validation respectful of the spiritual dimension of the patient. It would also provide the student with a closer approach with the patient. As Reginato et al. (2016) state, students learn not to dehumanize themselves, but do not know how to act in the face of a lesser humanistic education. Upon graduation, the physician must be prepared to meet not only physical needs, but also the emotional, sociocultural, and spiritual needs that occur in the lives of patients, meeting the biopsychosocial and spiritual paradigm.

In Brazil, 85% of the population has some religious affiliation, which leads us to envision patient acceptance when seeing their Spirituality/Religiosity considered in clinical care. However, only 10.4% of Brazilian medical schools offer the discipline Spirituality/Religiosity in their curriculum. In this article we have already cited some of the various publications related to the benefits that the approach of the spiritual dimension brings to patients. However, little has been published about what medical students in Brazil think about the insertion of Spirituality/Religiosity in clinical practice. By bringing spiritual anamnesis to the medical curriculum, we would provide students with a reflection on their own Spirituality/Religiosity. They would be prepared to deal with the pain and suffering of the patient providing a more humanized clinical care. These were the main reasons that leveraged the idealization of this research.

In this article, we will present the results obtained in the analysis of the questionnaire answered by medical students of the EPM/UNIFESP (from the first to the sixth year), Campus São Paulo, Brazil. The academic relevance of our research will be to present a general and quantitative overview about the insertion of “Spirituality/Religiosity in clinical practice” in the medical curriculum of the EPM. For the constitution of this purpose, we will investigate: (1) the importance that medical students of the EPM give to the spiritual dimension of the patient in clinical practice; (2) the credibility they give to the approach of the spiritual dimension of the patient as a therapeutic resource for the exercise of a more humanized clinical practice; (3) distancing from the patient’s suffering; (4) the agreement of the insertion of didactic-pedagogical actions in medical education related to the spiritual dimension of the patient. Do the religious affiliation and religiosity of the medical student interfere in their position? Is there a difference between the year of graduation in which the student is and his/her agreement to the propositions?

## Materials and Methods

### Ethics Committee and Study Design

This study was submitted to CEP/UNIFESP (Project No. 1556/2016), received the Certificate of Presentation for Ethical Appreciation (CAAE) no. 62062916.0.0000.5505 on the Brazil Platform, and was approved by the opinion embodied in CEP No. 1,855,656.

The study design presented a quantitative approach of the cross-sectional observational analytical type; several correlations were possible by submitting the collected data to statistical analysis. We chose to search for a greater sampling instead of a qualitative approach in order to measure, based on the conception of the study participants, the relevance of Spirituality/Religiosity as a therapeutic resource in clinical practice and the degree of agreement to the inclusion of didactic-pedagogical actions in medical education.

### Scenario and the Survey Participants

#### Scenario

The study scenario was the EPM at the Campus São Paulo, UNIFESP, Brazil. The choice of EPM as the setting of our research is consolidated by its excellence in the medical field. It is the largest federal medical education institution in Brazil, founded on June 1, 1933. It built a history marked by its pioneering spirit in the health area: (1) On September 30, 1936 laid the cornerstone of Hospital São Paulo (HSP), the first teaching hospital in the country; (2) created the Department of Medicine anticipating the departmental structure, which only came to be officially implemented in Brazilian higher education in 1965; (3) implemented Medical Residency Programs in Brazil, already in 1957; (4) created, from 1970, *stricto sensu* graduate programs, which form professors and researchers with a high level of technical-scientific competence, attributing to the EPM the highest rates of scientific productivity per teacher, at the national level.

#### Survey Participants

The participants were Medical School students, regularly enrolled in 2017, of the following cycles: basic (1st and 2nd years), professionalizing (3rd and 4th years) and internship (5th and 6th years).

We asked the Coordination of the Medical Course to indicate two volunteers from each class. We presented the data collection instrument for each pair and made relevant adjustments suggested by the students. All students were invited, and their participation was voluntarily upon completion of the Free and Informed Consent Form (TCLE). The 12 volunteers were responsible for inviting colleagues^4^, distributing, and returning the questionnaires and the Informed Consent to the researcher.

### Data Collection Instrument

A standardized questionnaire was developed for this study and applied in the classroom, containing 18 Likert-scale multiple-choice questions and one semi-open question:

#### Sociodemographic Profile (Questions 1 to 6)

Only questions 3 and 6 are reported in this article since the others (gender, ethnicity, age group, and family income) did not have significant results.

(3) Which undergraduate year are you enrolled in? (1). First year of Medical School; (2). Second year of Medical School; (3). Third year of Medical School; (4). Fourth year of Medical School; (5). Fifth year of Medical School; (6). Sixth year of Medical School.

(6) What is your religious affiliation? (1). Catholic; (2). Evangelical Protestant; (3). Spiritist; (4). Another. Which one? (5). No affiliation/Agnostic; (6). No affiliation/Atheist. (Semi-open question).

#### Duke University Religious Index (Questions 7 to 11)

The DUREL (Koenig et al., 1997; Moreira-Almeida et al., 2008) measures three of the main dimensions of Religiosity related to health outcomes. The scores in the three dimensions should be analyzed separately and the scores should not be summed.

##### Organizational Religiosity

(7) How often do you attend church, synagogue, or other religious meetings? (1). more than once a week; (2). once a week; (3). A few times a month; (4). a few times a year; (5). once a year or less; (6). never.

##### Non-organizational Religiosity

(8) How often do you spend time in private religious activities, such as prayer, meditation, or Bible study? (1). more than once a day; (2). daily; (3). twice or more times a week; (4). once a week; (5). a few times a month; (6). rarely or never.

##### Intrinsic Religiosity

*The following section contains three phrases about religious beliefs or experiences. Please note how much each sentence applies to you:*

(9) In my life, I experience the presence of the Divine: (1). definitely true; (2). it is generally true; (3). I am not sure; (4). in general it is not true; (5). definitely not true.

(10) My religious beliefs are what really lie behind my whole approach to life: (1). definitely true; (2). it is generally true; (3). I am not sure; (4). in general it is not true; (5). definitely not true.

(11) I try hard to carry my religion over into all other dealings in life: (1). definitely true; (2). it is generally true; (3). I am not sure; (4). in general it is not true; (5). definitely not true.

#### Spirituality/Religiosity in the Clinical and Academic Contexts (Questions 12 to 19)

Formulated propositions based on scientific literature and with the following alternatives: I fully agree, agree, indifferent, disagree and fully disagree.

(12) A physician should ask about spiritual questions that directly or indirectly influence a patient’s health positively.

(13) Regardless of their religious belief, a physician should respect a patient’s belief.

(14) A physician’s concern with a patient’s spiritual dimension demonstrates empathy and improves doctor-patient relationship.

(15) A physician should consider a patient’s emotional, sociocultural, and spiritual needs as well as their physical needs in the clinical practice.

(16) Care for the spiritual dimension is part of a patient’s comprehensive and humanized care.

(17) Denying his/her own emotions and distancing from a patient’s suffering is a defense strategy used by a physician.

(18) A good doctor should distance himself/herself from a patient’s suffering.

(19) The proposition of didactic-pedagogical actions related to a patient’s spiritual dimension in medical education is relevant.

### Statistical Analysis

The data were input in Excel 2016 for Windows spreadsheets for proper information storage. Initially, the statistical analysis of the collected information was performed descriptively, using absolute and relative frequencies (percentage).

The inferential analyses used to confirm or refute evidence found in the descriptive analysis were performed by the statistical programming language R, version 3.3.2, R Core Team, 2016. The inferential analysis was performed by Spearman coefficient (s) for correlation of ordinal variables and by Kruskal-Wallis test for analysis of religious affiliation (Siegel and Castellan, 2006). In all conclusions obtained through inferential analyses, the alpha significance level equal to 5% was used.

## Results

### Sociodemographic Profile

The study sample consisted of 72% of the total number of medical students from EPM, Campus São Paulo, UNIFESP, Brazil, regularly enrolled in the data collection period (from August to October 2017), with balanced participation among the three cycles of the medical program: 33% from the basic cycle (1st and 2nd years); 37% from the professionalizing cycle (3rd and 4th years); and 30% from the internship (5th and 6th years) (Table 1). The invitation made by the volunteers themselves to colleagues generated a high percentage of participants.

**TABLE 1:** Distribution of research participants by religious affiliation and cycle/undergraduate year.

	Religious Affiliation													
Cycle/Undergraduate Year		Catholic		Evangelical Protestant		Spiritist		Other		Not affiliated/Agnostic		Not affiliated/Atheist		TOTAL
		*n*	%	*n*	%	*n*	%	*n*	%	*n*	%	*n*	%	*n*
Basic	1^st^	27	27.5	16	16.3	9	9.2	4	4.1	23	23.5	19	19.4	98
	2^nd^	22	26.8	17	20.8	8	9.8	7	8.5	21	25.6	7	8.5	82
Professionalizing	3rd	27	29.3	10	10.9	9	9.8	3	3.3	31	33.7	12	13.0	92
	4^th^	28	25.7	18	16.5	14	12.9	8	7.3	30	27.5	11	10.1	109
Internship	5th	27	43.5	2	3.2	10	16.1	4	6.5	13	21.0	6	9.7	62
	6^th^	31	30.1	6	5.8	14	13.6	14	13.6	25	24.3	13	12.6	103
Total		162	29.7%	69	12.7%	64	11.7%	40	7.3%	143	26.2%	68	12.4%	546

Out of all study participants, 61.4% declared religious affiliation: 29.7% Catholicism; 12.7% Evangelical churches (63 Evangelicals, one Protestant, one Presbyterian, one Adventist and three Christians); 11.7% Spiritism (doctrine codified by Allan Kardec), 7.3% other religious denominations (Buddhism-10; belief in God-8, Judaism-6, Umbanda-3, The Church of Jesus Christ of Latter-day Saints-2, Shamanism-2, Hinduism-1, Isla-1, Seicho-No-Ie-1, Messianism-1, Taoism-1, Shinto-1, Tenrikyo-1, Deism-1, and Wicca-1) (Table 1).

In our sample, we noticed a pairing between the percentage of Spiritists (11.7%) and Evangelicals (12.7%). The distribution of students in different cycles of the medical program by religious affiliations (Table 1) showed that the percentage of those who declared themselves Spiritists and other religious denominations increased from the basic cycle to internship, while that of Evangelicals decreased.

Out of 38.6% students who did not follow any religious orientation, 26.2% declared themselves to be agnostic and 12.4% atheists. When dealing with religious affiliation, we chose to divide and name this group as “not affiliated/agnostic” and “not affiliated/atheist” (Table 1).

The results indicated that disbelief did not increase over the course of the medical studies; on the contrary, the percentage of those who declared themselves atheists was 38% in the basic cycle, 34% in the professionalizing cycle, and 28% in the internship.

Only one fifth-year student, out of 547 participants, did not answer this question.

### Duke University Religious Index

#### Organizational Religiosity

The measured Organizational Religiosity (OR) showed that 4.2% of the students attended religious institutions more than once a week, 10% once a week, 6.4% a few times a month, 27.6% a few times a year, 21.6% once a year or less, and 30.2% never attended (Table 2).

**TABLE 2:** Organizational Religiosity per undergraduate year.

	Attendance to Religious Institutions											
Undergraduate Year	more than once a week		once a week		a few times a month		a few times a year		once a year or less		never	
	*n*	%	*n*	%	*n*	%	*n*	%	*n*	%	*n*	%
1^st^	4	4.1	7	7.1	7	7.1	31	31.6	16	16.4	33	33.7
2^nd^	8	9.8	9	11.0	5	6.1	20	24.4	17	20.7	23	28.0
3^rd^	3	3.3	12	13.0	2	2.2	20	21.7	22	23.9	33	35.9
4^th^	4	3.7	15	13.8	11	10.0	36	33.0	21	19.3	22	20.2
5^th^	2	3.2	6	9.5	5	7.9	14	22.2	19	30.2	17	27.0
6^th^	2	2.0	6	5.8	5	4.9	30	29.1	23	22.3	37	35.9
Total	23	4.2%	55	10.0%	35	6.4%	151	27.6%	118	21.6%	165	30.2%

Considering that “more than once a week,” “once a week,” and “a few times a month” are positive responses to the religious institution’s frequency, we have an approximate percentage of 18.3% in the 1st year, 26.9% in the 2nd year, 18.5% in the 3rd year, 27.5% in the 4th year, 20.6% in the 5th year, and 12.7% in the 6th year (Table 2).

Evangelicals had the highest religious frequency. Most Catholic, Spiritist, and other denomination students attend religious institutions “a few times a year.” Agnostics focus between “once a year or less” and “never,” and most atheists in “never” (Table 3).

**TABLE 3:** Attendance to religious institution by students from different religious affiliations.

	Religious Affiliation													
Attendance to religious institution	Catholic		Evangelical Protestant		Spiritist		Other		Not affiliated/Agnostic		Not affiliated/Atheist		Total	
	*n*	%	*n*	%	*n*	%	*n*	%	*n*	%	*n*	%	*n*	%
more than once a week	4	2.5	17	24.6	0	0	2	5.0	0	0	0	0	23	4.0%
once a week	23	14.2	25	36.2	5	7.8	2	5.0	0	0	0	0	55	10.0%
a few times a month	12	7.4	11	15.9	6	9.4	6	15.0	0	0	0	0	35	6.4%
a few times a year	64	39.5	11	15.9	30	46.9	17	42.5	24	16.8	4	5.9	150	27.5%
once a year or less	36	22.2	5	7.2	15	23.4	7	17.5	47	32.9	8	11.8	118	21.6%
never	23	14.2	0	0	8	12.5	6	15.0	72	50.3	56	82.4	165	30.5%

#### Non-organizational Religiosity

Students’ Non-organizational Religiosity (NOR) was measured by time dedicated to individual religious activities: 4.6% dedicate themselves to this practice more than once a day, 17.5% daily, 7.9% twice or more times a week, 6.2% once a week, 17% a few times a month, and 46.8% rarely or never (Table 4).

**TABLE 4:** Non-organizational Religiosity per undergraduate year.

	Time dedicated to individual religious activities											
Undergraduate Year	more than once a day		Daily		twice or more times a week		once a week		a few times a month		rarely or never	
	*n*	%	*n*	%	*n*	%	*n*	%	*n*	%	*n*	%
1^st^	4	4.1	21	21.4	10	10.2	5	5.1	16	16.4	42	42.8
2^nd^	5	6.1	14	17.1	6	7.3	3	3.7	11	13.4	43	52.4
3^rd^	6	6.5	10	10.9	6	6.5	4	4.3	16	17.4	50	54.4
4^th^	7	6.4	18	16.5	8	7.3	11	10.1	24	22.0	41	37.7
5^th^	1	1.6	16	25.4	7	11.1	1	1.6	8	12.7	30	47.6
6^th^	2	2.0	17	16.5	6	5.8	10	9.7	18	17.5	50	48.5
Total	25	4.6%	96	17.5%	43	7.9%	34	6.2%	93	17.0%	256	46.8%

Since “rarely or never” is the only answer considered negative for Non-organizational Religiosity, we have an approximate positive percentage of 57.2% in the 1st year, 47.6% in the 2nd year, 45.6% in the 3rd year, 62.3% in the 4th year, 52.4% in the 5th year, and 51.5% in the 6th year (Table 4).

Among religious affiliations, 59.4% of Evangelicals dedicate themselves to individual religious activities at least once a day, followed by 32.1% of Catholics. Dedication of at least once a week to these activities was reported by 45.4% of Spiritists and 42.5% of students from other religious affiliations (Table 5).

**TABLE 5:** Time dedicated to individual religious activities by students from different religious affiliations.

	Religious Affiliation													
Time dedicated to individual religious activities	Catholic		Evangelical Protestant		Spiritist		Other		Not affiliated/Agnostic		Not affiliated/Atheist		Total	
	*n*	%	*n*	%	*n*	%	*n*	%	*n*	%	*n*	%	*n*	%
more than once a day	7	4.3	11	15.9	1	1.6	3	7.5	3	2.0	0	0.0	25	4.6%
Daily	45	27.8	30	43.5	7	11.0	8	20.0	6	4.0	0	0.0	96	17.6%
twice or more times a week	18	11.1	7	10.1	9	14.1	3	7.5	4	3.0	2	3.0	43	7.9%
once a week	10	6.2	5	7.3	12	18.7	3	7.5	4	3.0	0	0.0	34	6.2%
few times a month	36	22.2	10	14.5	15	23.4	12	30.0	16	11.0	3	4.0	92	16.8%
rarely or never	46	28.4	6	8.7	20	31.2	11	27.5	110	77.0	63	93.0	256	46.9%

#### Intrinsic Religiosity

Measurement of the centrality of transcendent in students’ lives showed very strong (answer - definitely true) Intrinsic Religiosity (IR) in 13%; strong (answer - it is generally true) in 20.6%, moderate (answer - I am not sure) in 21.6%, weak (answer - in general it is not true) in 18.5%, and very weak (answer - definitely not true) in 26.3% (Table 6).

**TABLE 6:** Students’ Intrinsic Religiosity per undergraduate year.

	Centrality of religion in life									
Undergraduate Year	very strong		strong		moderate		weak		very weak	
	n	%	n	%	n	%	n	%	n	%
1^st^	13	13.3	17	17.3	23	23.5	18	18.4	27	27.5
2^nd^	14	17.1	13	15.9	18	21.9	15	18.3	22	26.8
3^rd^	9	9.8	18	19.6	15	16.3	21	22.8	29	31.5
4^th^	20	18.3	27	24.8	21	19.3	20	18.3	21	19.3
5^th^	8	12.7	15	23.8	16	25.4	8	12.7	16	25.4
6^th^	7	6.8	23	22.3	25	24.3	19	18.4	29	28.2
Total	71	13.0%	113	20.6%	118	21.6%	101	18.5%	144	26.3%

Considering “definitely true” and “it is generally true” as positive responses to Intrinsic Religiosity, we have an approximate percentage of 30.6% in the 1st year, 33% in the 2nd year, 29.4% in the 3rd year, 43.1% in the 4th year, 36.5% in the 5th year, 29.1% in the 6th year (Table 6).

Most of the Evangelicals (82.6%) showed religious centrality ranging from strong to very strong. On the other hand, 63% of Catholics, 62.5% of Spiritists, and 67.5% of other religious denominations varied from centrality moderate to strong (Table 7).

**TABLE 7:** Centrality of religion in the lives of students from different religious affiliations.

	Religious Affiliation													
Centrality of Religion in life	Catholic		Evangelical Protestant		Spiritist		Other		Not affiliated/Agnostic		Not affiliated/Atheist		Total	
	*n*	%	*n*	%	*n*	%	*n*	%	*n*	%	*n*	%	*n*	%
very strong	23	32.4	32	45.1	8	11.3	5	7.0	3	4.2	0	0	71	13.0%
strong	53	46.9	25	22.1	17	15.1	11	9.7	6	5.3	1	0.9	113	20.7%
moderate	49	41.9	8	6.8	23	19.7	16	13.7	18	15.4	3	2.5	117	21.4%
weak	28	27.7	4	4.0	10	9.9	7	6.9	46	45.6	6	5.9	101	18.5%
very weak	9	6.2	0	0	6	4.2	1	0.7	70	48.6	58	40.3	144	26.4%

#### Inferential Analysis - Duke University Religious Index

The Kruskal–Wallis test (Table 8) showed a significant difference between “Religious Affiliation” and the three aspects of DUREL. Organizational Religiosity (OR), Non-Organizational Religiosity (NOR) and Intrinsic Religiosity (IR) maintained the same pattern of behavior, presented high scores among students with religious affiliation; low scores among agnostics; and non-existent among atheists.

**TABLE 8:** Kruskal–Wallis test - religious affiliation and students’ religiosity Duke University Religious Index (DUREL).

Kruskal–Wallis		DUREL					
religion versus		7	8	9	10	11	media

*p*-value		0.000	0.000	0.000	0.000	0.000	0.000

Ranks							

	Affiliation	N	Mean Rank		Affiliation	N	Mean Rank
OR 7	1	162	226.66	IR 10	1	162	218.73
	2	69	90.16		2	69	118.22
	3	64	234.93		3	64	240.43
	4	40	223.70		4	40	189.86
	5	143	371.26		5	143	373.49
	6	68	431.15		6	68	431.60
	Total	546			Total	546	
NOR 8	1	162	221.24	IR 11	1	162	229.48
	2	69	133.51		2	69	117.72
	3	64	250.47		3	64	206.27
	4	40	228.45		4	40	184.85
	5	143	361.71		5	143	381.10
	6	68	402.72		6	68	425.60
	Total	546			Total	546	
IR 9	1	162	189.02	IR media	1	162	205.55
	2	69	127.27		2	69	107.30
	3	64	243.05		3	64	224.38
	4	40	239.64		4	40	198.68
	5	143	369.85		5	143	381.19
	6	68	469.10		6	68	467.82
	Total	546			Total	546	

According to the estimates of Spearman’s Correlation Coefficient (Table 9), we confirmed a statistically significant positive correlation between all questions of the Duke Religiosity Index among themselves, that is, when we observed an increase in one variable (OR, NOR and IR), simultaneously there was an increase in the other compared variable (OR, NOR and IR).

**TABLE 9:** Spearman coefficient (s) – undergraduate year and Duke University Religious Index (DUREL).

Spearman’s rho			Undergr Year	OR	NOR	IR			
				7	8	9	10	11	media
Undergraduate Year		Correlation Coefficient	1.000	0.033	0.015	−0.022	−0.050	0.028	−0.013
		Sig. (2-tailed)	.	0.442	0.728	0.610	0.240	0.521	0.758
		N	547	547	547	547	547	547	547
OR	7	Correlation Coefficient	0.033	1.000	0.637	0.655	0.672	0.709	0.728
		Sig. (2-tailed)	0.442	.	0.000	0.000	0.000	0.000	0.000
		N	547	547	547	547	547	547	547
NOR	8	Correlation Coefficient	0.015	0.637	1.000	0.649	0.660	0.714	0.720
		Sig. (2-tailed)	0.728	0.000	.	0.000	0.000	0.000	0.000
		N	547	547	547	547	547	547	547
IR	9	Correlation Coefficient	−0.022	0.655	0.649	1.000	0.816	0.743	0.918
		Sig. (2-tailed)	0.610	0.000	0.000	.	0.000	0.000	0.000
		N	547	547	547	547	547	547	547
	10	Correlation Coefficient	−0.050	0.672	0.660	0.816	1.000	0.865	0.953
		Sig. (2-tailed)	0.240	0.000	0.000	0.000	.	0.000	0.000
		N	547	547	547	547	547	547	547
	11	Correlation Coefficient	0.028	0.709	0.714	0.743	0.865	1.000	0.921
		Sig. (2-tailed)	0.521	0.000	0.000	0.000	0.000	.	0.000
		N	547	547	547	547	547	547	547
	media	Correlation Coefficient	−0.013	0.728	0.720	0.918	0.953	0.921	1.000
		Sig. (2-tailed)	0.758	0.000	0.000	0.000	0.000	0.000	.
		N	547	547	547	547	547	547	547

*Undergr Year, Undergraduate Year; OR, Organizational Religiosity (question 7); NOR, Non-organizational Religiosity (question 8); IR, Intrinsic Religiosity (questions 9, 10, 11 - media).*

### Spirituality/Religiosity in Clinical and Academic Contexts

We will present the data in two separate tables (Tables 10, 11) so that they can be included in the body of the article.

**TABLE 10:** Propositions regarding Spirituality/Religiosity in the clinical context.

Propositions	Undergraduate Year	I fully agree		Agree		Indifferent		Disagree		I strongly disagree		Total
		*n*	%	*n*	%	*n*	%	*n*	%	*n*	%	*n*
(12) A physician should ask about spiritual questions that directly or indirectly influence a patient’s health positively.	1st	17	17.3	49	50.0	20	20.4	7	7.2	5	5.1	98
	2^nd^	25	30.5	45	54.9	9	11.0	3	3.6	0	0	82
	3^rd^	25	27.2	52	56.5	12	13.0	2	2.2	1	1.1	92
	4^th^	37	33.9	50	45.9	16	14.7	5	4.6	1	0.9	109
	5^th^	22	34.9	30	47.6	8	12.7	2	3.2	1	1.6	63
	6^th^	28	27.2	54	52.4	13	12.6	5	4.9	3	2.9	103
	Total	154	28.1%	280	51.2%	78	14.3%	24	4.4%	11	2.0%	547
(13) Regardless of their religious belief, a physician should respect a patient’s belief.	1st	86	87.8	11	11.2	1	1.0	0	0	0	0	98
	2nd	68	83.0	12	14.6	0	0	2	2.4	0	0	82
	3rd	79	85.9	10	10.8	1	1.1	1	1.1	1	1.1	92
	4th	101	92.7	8	7.3	0	0	0	0	0	0	109
	5th	52	82.5	10	15.9	0	0	1	1.6	0	0	63
	6th	85	82.5	17	16.5	1	1.0	0	0	0	0	103
	Total	471	86.1%	68	12.5%	3	0.5%	4	0.7%	1	0.2%	547
(14) A physician’s concern with a patient’s spiritual dimension demonstrates empathy and improves doctor-patient relationship.	1st	46	47.0	40	40.8	10	10.2	1	1.0	1	1.0	98
	2nd	46	56.1	32	39.0	4	4.9	0	0.0	0	0.0	82
	3rd	47	51.1	38	41.3	5	5.4	2	2.2	0	0.0	92
	4^th^	72	66.1	34	31.2	2	1.8	1	0.9	0	0.0	109
	5^th^	40	63.5	19	30.1	2	3.2	2	3.2	0	0.0	63
	6^th^	52	50.5	40	38.8	8	7.8	3	2.9	0	0.0	103
	Total	303	55.4%	203	37.1%	31	5.7%	9	1.7%	1	0.1%	547
(15) A physician should consider a patient’s emotional, sociocultural, and spiritual needs as well as their physical needs in the clinical practice.	1st	52	54.8	40	42.1	2	2.1	1	1.0	0	0	95
	2^nd^	61	74.4	19	23.2	1	1.2	1	1.2	0	0	82
	3^rd^	65	71.4	26	28.6	0	0.0	0	0.0	0	0	91
	4^th^	74	68.5	29	26.9	4	3.7	1	0.9	0	0	108
	5^th^	38	61.3	21	33.9	2	3.2	1	1.6	0	0	62
	6^th^	63	61.2	37	35.9	2	1.9	1	1.0	0	0	103
	Total	353	65.3%	172	31.8%	11	2.0%	5	0.9%	0	0	541

**TABLE 11:** Propositions related to Spirituality/Religiosity in clinical and academic contexts.

Propositions	Year of Graduation	I fully agree		Agree		Indifferent		Disagree		I strongly disagree		Total
		*n*	%	*n*	%	*n*	%	*n*	%	*n*	%	*n*
(16) Care for the spiritual dimension is part of a patient’s comprehensive and humanized care.	1st	36	36.7	43	43.9	14	14.3	4	4.1	1	1.0	98
	2^nd^	31	37.8	41	50.0	7	8.5	3	3.7	0	0.0	82
	3^rd^	43	46.8	36	39.1	8	8.7	5	5.4	0	0.0	92
	4^th^	52	47.7	44	40.4	7	6.4	6	5.5	0	0.0	109
	5^th^	26	42.0	24	38.7	8	12.9	3	4.8	1	1.6	62
	6^th^	39	37.9	42	40.8	12	11.6	8	7.8	2	1.9	103
	Total	227	41.6%	230	42.1%	56	10.3%	29	5.3%	4	0.7%	546
(17) Denying his/her own emotions and distancing from a patient’s suffering is a defense strategy used by a physician.	1st	5	5.1	49	50.0	13	13.3	23	23.5	8	8.1	98
	2^nd^	7	8.5	42	51.2	11	13.4	18	22.0	4	4.9	82
	3^rd^	11	12.0	49	53.3	14	15.2	17	18.5	1	1.0	92
	4^th^	18	16.5	55	50.5	17	15.6	16	14.7	3	2.7	109
	5^th^	11	17.7	38	61.3	5	8.1	7	11.3	1	1.6	62
	6^th^	18	17.5	55	53.4	14	13.6	13	12.6	3	2.9	103
	Total	70	12.8%	288	52.8%	74	13.5%	94	17.2%	20	3.7%	546
(18) A good doctor should distance himself/herself from a patient’s suffering.	1st	0	0.0	1	1.0	5	5.1	74	75.5	18	18.4	98
	2^nd^	0	0.0	0	0.0	4	4.9	45	54.9	33	40.2	82
	3^rd^	0	0.0	0	0.0	8	8.7	55	59.8	29	31.5	92
	4^th^	2	1.8	3	2.8	8	7.3	70	64.2	26	23.9	109
	5^th^	0	0.0	2	3.2	5	8.1	44	71.0	11	17.7	62
	6^th^	0	0.0	2	1.9	12	11.7	57	55.3	32	31.1	103
	Total	2	0.4%	8	1.5%	42	7.7%	345	63.2%	149	27.2%	546
(19) The proposition of didactic-pedagogical actions related to a patient’s spiritual dimension in medical education is relevant.	1st	12	12.2	44	44.9	31	31.6	7	7.2	4	4.1	98
	2^nd^	16	19.5	39	47.6	21	25.6	5	6.1	1	1.2	82
	3^rd^	12	13.2	41	45.0	26	28.6	11	12.1	1	1.1	92
	4^th^	14	12.9	60	55.1	26	23.8	7	6.4	2	1.8	109
	5^th^	7	11.3	28	45.2	19	30.6	6	9.7	2	3.2	62
	6^th^	17	16.5	46	44.7	26	25.2	10	9.7	4	3.9	103
	Total	78	14.3%	258	47.4%	149	27.3%	46	8.4%	14	2.6%	545

#### Spirituality/Religiosity in Clinical Context (Propositions 12 to 15)

There was agreement in propositions 12 to 15 of 79.3, 98.6, 92.5, and 97.1%, respectively, which suggests that students consider: the relevance of spiritual anamnesis, importance of respect for a patient’s religious beliefs, use of this practice to demonstrate empathy and improve doctor-patient relationship, and the relevance of considering a patient’s emotional, sociocultural, and spiritual needs in the clinical practice (Table 10).

#### Spirituality/Religiosity in Clinical and Academic Contexts (Propositions 16 to 19)

Propositions 16 and 17 had an agreement of 83.7% and 65.6%, respectively. There was a 90.4% disagreement for the proposition “A good doctor should distance himself/herself from a patient’s suffering.” Only 11% of the students are opposed to the proposition of didactic-pedagogical actions in medical education related to a patient’s spiritual dimension while 61.7% consider it relevant and 27.3% are indifferent (Table 11).

#### Inferential Analysis in Clinical and Academic Contexts

The Kruskal–Wallis test (Table 12) showed a significant difference between “Religious Affiliation” and propositions 14 and 16 of the clinical context – in both there was greater agreement between students who have religious affiliation and among agnostics, with lower agreement among atheists. The Kruskal–Wallis test (Table 12) showed a significant difference between “Religious Affiliation” and the purpose 19 of the academic context – there was greater agreement among students who have religious affiliation; lower among agnostics; and, among atheists.

**TABLE 12:** Kruskal–Wallis test - clinical and academic contexts.

Kruskal–Wallis	Clinical and Academic Contexts - Questions							
religion versus	12	13	14	15	16	17	18	19
*p*-value	0.227	0.137	0.013	0.707	0.038	0.121	0.877	0.003

Ranks							

	Affiliation	N	Mean Rank		Affiliation	N	Mean Rank
12	1	162	258.08	16	1	162	246.06
	2	69	292.01		2	69	280.39
	3	64	268.01		3	64	293.57
	4	40	266.56		4	40	262.41
	5	143	270.42		5	143	277.92
	6	68	307.18		6	68	310.20
	Total	546			Total	546	
13	1	162	269.43	17	1	162	294.20
	2	69	266.69		2	69	271.24
	3	64	273.33		3	64	238.84
	4	40	262.40		4	40	243.46
	5	143	270.15		5	143	274.10
	6	68	303.85		6	68	275.49
	Total	546			Total	546	
14	1	162	264.95	18	1	162	279.54
	2	69	295.11		2	69	281.41
	3	64	276.05		3	64	268.91
	4	40	230.18		4	40	286.31
	5	143	262.09		5	143	265.40
	6	68	319.02		6	68	264.89
	Total	546			Total	546	
15	1	162	279.61	19	1	162	257.20
	2	69	263.74		2	69	267.24
	3	64	265.87		3	64	265.23
	4	40	263.39		4	40	232.90
	5	143	268.08		5	143	280.56
	6	68	293.37		6	68	335.50
	Total	546			Total	546	

Spearman’s Correlation Coefficient (Table 13) confirmed a statistically significant negative correlation between the year of graduation in which the student is present and the proposition 12 (*S* = −0.085; *p* = 0.046) - as the student approaches his/her academic background, the agreement with the assertive increases. Spearman’s Correlation Coefficient (Table 13) confirmed a statistically significant negative correlation between the year of graduation in which the student is present and the proposition 17 (*S* = −0.180; *p* = 0.000) - as the student approaches his/her academic background, the agreement with the assertive increases.

**TABLE 13:** Spearman coefficient (s) – undergraduate year/DUREL and clinical/academic contexts.

Spearman’s rho			Clinical and Academic Contexts - Questions							
			12	13	14	15	16	17	18	19
Undergraduate Year		Correlation Coefficient	−0.085	0.024	−0.050	0.019	−0.001	−0.180	−0.043	−0.002
		Sig. (2-tailed)	0.046	0.577	0.245	0.659	0.988	0.000	0.311	0.971
		N	547	547	547	547	547	547	547	547
OR	7	Correlation Coefficient	0.069	0.065	0.101	0.096	0.127	−0.016	−0.099	0.153
		Sig. (2-tailed)	0.107	0.131	0.018	0.025	0.003	0.704	0.020	0.000
		N	547	547	547	547	547	547	547	547
NOR	8	Correlation Coefficient	0.061	0.026	0.047	0.073	0.118	0.003	−0.053	0.139
		Sig. (2-tailed)	0.155	0.539	0.272	0.087	0.006	0.953	0.212	0.001
		N	547	547	547	547	547	547	547	547
IR	9	Correlation Coefficient	0.118	0.099	0.150	0.117	0.202	−0.018	−0.106	0.233
		Sig. (2-tailed)	0.006	0.020	0.000	0.006	0.000	0.679	0.013	0.000
		N	547	547	547	547	547	547	547	547
	10	Correlation Coefficient	0.110	0.088	0.154	0.127	0.173	−0.002	−0.081	0.210
		Sig. (2-tailed)	0.010	0.040	0.000	0.003	0.000	0.962	0.059	0.000
		N	547	547	547	547	547	547	547	547
	11	Correlation Coefficient	0.096	0.071	0.139	0.136	0.138	0.032	−0.069	0.208
		Sig. (2-tailed)	0.025	0.097	0.001	0.001	0.001	0.459	0.108	0.000
		N	547	547	547	547	547	547	547	547
	media	Correlation Coefficient	0.119	0.096	0.160	0.133	0.185	0.006	−0.090	0.235
		Sig. (2-tailed)	0.005	0.025	0.000	0.002	0.000	0.891	0.035	0.000
		N	547	547	547	547	547	547	547	547

*Legends: OR, Organizational Religiosity (question 7); NOR, Non-organizational Religiosity (question 8); IR, Intrinsic Religiosity (questions 9, 10, 11 - media).*

Spearman’s Correlation Coefficient (Table 13) confirmed a statistically significant negative correlation between OR (*S* = −0.0099; *p* = 0.020) and the proposition 18 - the higher the Organizational Religiosity of the students, the lower the agreement with the proposition. Spearman’s Correlation Coefficient (Table 13) confirmed a statistically significant negative correlation between IR (*S* = −0.0090; *p* = 0.035) and the proposition 18 - the higher the Intrinsic Religiosity of the students, the lower the agreement with the proposition.

Spearman’s Correlation Coefficient (Table 13) confirmed a statistically significant positive correlation between OR and the proposition 14 (*S* = 0.101; *p* = 0.018) - the higher the Organizational Religiosity, the higher the students’ degree of agreement to this assertion. Spearman’s Correlation Coefficient (Table 13) confirmed a statistically significant positive correlation between OR and the proposition 15 (*S* = 0.096; *p* = 0.025) - the higher the Organizational Religiosity, the higher the students’ degree of agreement to this assertion. Spearman’s Correlation Coefficient (Table 13) confirmed a statistically significant positive correlation between IR and the questions 12, 14, and 15 of the clinical context - the higher the Intrinsic Religiosity, the higher the degree of agreement of the students to the questions related to Spirituality/Religiosity in the clinical and academic contexts.

Spearman’s Correlation Coefficient (Table 13) confirmed a statistically significant positive correlation between DUREL and the proposition 16 [OR (*S* = 0.127; *p* = 0.003), NOR (*S* = 0.118; *p* = 0.006) and IR (*S* = 0.185; *p* = 0.000)] - the higher the DUREL scores, the higher the students’ degree of agreement to this question. Spearman’s Correlation Coefficient (Table 13) confirmed a statistically significant positive correlation between DUREL and the proposition 19 [OR (*S* = 0.153; *p* = 0.000); NOR (*S* = 0.139; *p* = 0.001); and IR (*S* = 0.235; *p* = 0.000)] - the higher the DUREL scores, the higher the degree of agreement of the students to this question.

Spearman’s Correlation Coefficient (Table 14) confirmed statistically significant negative correlation between propositions 18 and 14 (*S* = −0.163; *p* = 0.000) - the higher the agreement of the students with the question 14, the lower the agreement with the proposition 18. Spearman’s Correlation Coefficient (Table 14) confirmed statistically significant negative correlation between propositions 18 and 15 (*S* = −0.154; *p* = 0.000) - the higher the agreement of the students with the question 15, the lower the agreement with the proposition 18. Spearman’s Correlation Coefficient (Table 14) confirmed statistically significant negative correlation between propositions 18 and 16 (*S* = −0.152; *p* = 0.000) - the higher the agreement of the students with the question 16, the lower the agreement with the proposition 18. Spearman’s Correlation Coefficient (Table 14) confirmed statistically significant negative correlation between propositions 18 and 19 (*S* = −0.157; *p* = 0.000) - the higher the agreement of the students with the question 19, the lower the agreement with the proposition 18.

**TABLE 14:** Spearman coefficient (s) – clinical and academic contexts.

Spearman’s rho			Clinical and Academic Contexts							
			12	13	14	15	16	17	18	19
Clinical And Academic Contexts	12	Correlation Coefficient	1.000	0.151	0.497	0.333	0.466	0.057	−0.052	0.343
		Sig. (2-tailed)	.	0.000	0.000	0.000	0.000	0.185	0.220	0.000
		N	547	547	547	547	547	547	547	547
	13	Correlation Coefficient	0.151	1.000	0.254	0.230	0.197	0.021	−0.071	0.129
		Sig. (2-tailed)	0.000	.	0.000	0.000	0.000	0.630	0.099	0.002
		N	547	547	547	547	547	547	547	547
	14	Correlation Coefficient	0.497	0.254	1.000	0.375	0.527	0.049	−0.163	0.408
		Sig. (2-tailed)	0.000	0.000	.	0.000	0.000	0.256	0.000	0.000
		N	547	547	547	547	547	547	547	547
	15	Correlation Coefficient	0.333	0.230	0.375	1.000	0.435	0.105	−0.154	0.312
		Sig. (2-tailed)	0.000	0.000	0.000	.	0.000	0.014	0.000	0.000
		N	547	547	547	547	547	547	547	547
	16	Correlation Coefficient	0.466	0.197	0.527	0.435	1.000	0.029	−0.152	0.457
		Sig. (2-tailed)	0.000	0.000	0.000	0.000	.	0.503	0.000	0.000
		N	547	547	547	547	547	547	547	547
	17	Correlation Coefficient	0.057	0.021	0.049	0.105	0.029	1.000	0.072	0.053
		Sig. (2-tailed)	0.185	0.630	0.256	0.014	0.503	.	0.093	0.214
		N	547	547	547	547	547	547	547	547
	18	Correlation Coefficient	−0.052	−0.071	−0.163	−0.154	−0.152	0.072	1.000	−0.157
		Sig. (2-tailed)	0.220	0.099	0.000	0.000	0.000	0.093	.	0.000
		N	547	547	547	547	547	547	547	547
	19	Correlation Coefficient	0.343	0.129	0.408	0.312	0.457	0.053	−0.157	1.000
		Sig. (2-tailed)	0.000	0.002	0.000	0.000	0.000	0.214	0.000	.
		N	547	547	547	547	547	547	547	547

Spearman’s Correlation Coefficient (Table 14) confirmed statistically significant positive correlation between questions in the clinical context 12 to 16. Spearman’s Correlation Coefficient (Table 14) confirmed statistically significant positive correlation between questions in the clinical context 12 to 16 with proposition 19 of the academic context - the greater the agreement of the students with the related propositions, the greater the agreement with proposition 19.

## Discussion

There are several theories used by scientists of religion. Hanegraaff (1999) established, in a threefold scheme, the difference between religion and spirituality: *religion* (general), *a religion* (specific) and *a spirituality*. The author presents a subtle difference when it comes to defining Religion in general and *a* specific *religion*. *Religion* would be “any symbolic system,” while *A Religion* would be “a symbolic system, embedded in a social institution,” both influence human actions and allow “to maintain ritualistic contact between the everyday world and a more general meta-empirical picture of meanings.” *A spirituality* is defined as *“any* human practice that maintains contact between the everyday world and a more general meta-empirical picture of meanings through the individual manipulation of symbolic systems” (Hanegraaff, 1999, p. 371–372).

Asad (2010) problematizes the idea of an anthropological definition of religion by referring this effort to a particular history of knowledge and power, including a particular understanding of our legitimate past and future, from which the modern world was built. It states that many of the theories about religion come from a modern Western model, which imprints on religion a trans-historical and cross-cultural character. It argues that there cannot be a universal definition of religion, not only because its constituent elements and its relationships are historically specific, but because this definition is itself the historical product of discursive processes.

In 1998, the World Health Organization (World Health Organization [WHO], 1998) presented a proposal for the definition of religion, religiosity, and spirituality. In the WHO definition, Religion would be “the belief in the existence of a dominant supernatural power, creator and controller of the universe, which gave the human being a spiritual nature that continues to exist after the death of the body,” while Religiosity would be the act of following, practicing, or believing in a religion. With reference to the definition of Spirituality, who says it would be “[.] the belief in a non-material nature with the assumption that there is more to life than what can be perceived or fully understood.” He adds that “Spirituality addresses issues such as the meaning of life and purpose in life and is not necessarily limited to any specific types of beliefs or practices” (World Health Organization [WHO], 1998, p. 7).

Stern (2017) states that the differentiation between religion, religiosity and spirituality is very difficult to trace and points out several problems in the WHO definitions from the perspective of the scholar of religion. Initially, the definitions of religion and religiosity are based exclusively on the model of Abrahamic religions. By using the term “Spirituality,” which would not necessarily relate to any religion, the WHO supports physicians to act in this field without hurting their codes of ethics but does not identify elements that can be only “spiritual,” without being “religious.”

The scientific literature presents a wide variety of works that deal with the inclusion of the Discipline of Spirituality and Religiosity in the medical curriculum. However, in pedagogical projects there is no specification of the most indicated professional category to coordinate this course and teach the classes. Stern (2018) believes that it is possible, with the application of concepts from the study of religion, to build professional bridges between study of religion and health professionals. By the specific training in the theme and acquired skills, the scholar of religion has the most appropriate profile to train medical students in Spirituality/Religiosity.

We agree that academic training does not empower physicians, future teachers, to use the spiritual/religious context in clinical practice, therefore, they will not be able to transmit to their students a knowledge they do not have. It is necessary that a multidisciplinary team act, led by a scholar of religion, who can teach students the bases of religious traditions, so diverse in Brazil, so that when they receive, for example, a patient who is a Jehovah’s Witness know that they will not be able to perform blood transfusion without authorization. In the prescription of medications, other religions have restrictions on substances, days of the week, schedules. The scholar of religion will be able to guide students on how to detect spiritual suffering and approach the patient about wanting to talk to the chaplain or, if not, with the religious leader of his religious belief. The doctor can work together with the scholar of religion to explain to the students how to proceed with the taking of spiritual history. Validated simple instruments, as FICA [F (Faith/belief)/I (Importance or influence)/C (Community)/A (Action in treatment)] (Puchalski and Romer, 2000) and HOPE [H (Sources of Hope)/O (Organized Religion)/P (Personal spirituality and practice)/E (Effects on medical treatment and terminal matters)] (Anandarajah and Hight, 2001), for example, can detect, in a matter of minutes, if the patient needs spiritual care, not for the doctor to treat him, but to refer him to the qualified professional.

Medicine is a compassionate and selfless service profession and, increasingly, the field recognizes the need to integrate training in Spirituality in patient care as part of medical education (Puchalski and Larson, 1998; Puchalski, 2001b; Lucchetti et al., 2012; Reginato et al., 2016). Barnett and Fortin (2006) found that both medical undergraduates and residents changed their attitudes regarding their appreciation of the approach of a patient’s spiritual dimension in medical anamnesis after adequate training. It is considered that, through repeated and varied exposure, students will be able to build a more positive attitude toward the importance attributed to the spiritual approach in clinical practice. Knowledge brings security and Spirituality is no longer considered dependent on religious affiliation, conviction, or practice. By acquiring confidence in the taking of spiritual history, they begin to value it and consider it beneficial to patients (Chibnall and Duckro, 2000; Chibnall and Brooks, 2001; Chibnall et al., 2002; Fortin and Barnett, 2004; Anandarajah and Mitchell, 2007; Culliford, 2009).

We emphasize that with the appropriate training in Spirituality/Religiosity, the main obstacles pointed out by physicians to take a patient’s spiritual history can be overcome. Training should lead them to: (1) acquire a more positive attitude toward the importance attributed to the spiritual approach in clinical practice; (2) have a better understanding of the role of Spirituality in Health; (3) know the foundations of the main religious traditions in order to acquire subsidies to respect patients’ beliefs and understand their spiritual needs; (4) develop spiritual care skills to care for patients from different cultures and different spiritual and religious contexts; (5) acquire knowledge on the philosophy of care; (6) be sensitized to a patient’s spiritual and cultural needs; (7) recognize a patient’s spiritual suffering and promote comprehensive care; (8) be naturally receptive and safe to support and encourage patients’ religious beliefs; (9) learn that more than 3,300 scientific studies on Spirituality/Religiosity and Health have proven that religious activities and beliefs are related to better physical and mental health, and quality of life in the most different aspects; (10) explore the relevant instruments published in the literature that guide the taking of a patient’s spiritual history; (11) take the spiritual history during the anamnesis, preferably at the end of social history; (12) practice doing spiritual anamnesis a few times to be able to carry it out in a few minutes; (13) become aware of their own finitude, and understand death as a natural process of life (Chibnall and Duckro, 2000; Chibnall et al., 2002; Graves et al., 2002; Sandor et al., 2006; Anandarajah and Mitchell, 2007; Feldstein et al., 2008; Culliford, 2009; Lucchetti et al., 2010, 2013a,^2015^; Koenig, 2012a,b, 2015; Koenig et al., 2012; Kübler-Ross, 2012; Lucchetti and Lucchetti, 2014; Talley and Magie, 2014; Arantes, 2016; Cavalcante et al., 2016; Moreira-Almeida et al., 2016; Peres et al., 2018; Puchalski et al., 2020).

It is noteworthy to mention that, in the medical area, the therapeutic use of a patient’s spiritual dimension in Palliative Care stands out (D’Alessandro et al., 2020; Puchalski et al., 2020). Other areas, such as Psychiatry and Cardiology, have also shown interest in their patients’ spiritual dimension. The *Position Statement on Spirituality and Religion in Psychiatry*, proposed by the Religion, Spirituality and Psychiatry Section of the World Psychiatric Association (WPA), was published, and approved by the WPA executive committee in September 2015 (Moreira-Almeida et al., 2016). In Cardiology, Spirituality is highlighted in the *Updated Cardiovascular Prevention Guideline of the Brazilian Society of Cardiology*, published in 2019, including a new chapter entitled *Spirituality and Psychosocial Factors in Cardiovascular Medicine* (Avezum and Esporcatte, 2019). The *Prevention Guidelines of the Brazilian Society of Cardiology* guide physicians – and health professionals in general – on how to better address spiritual issues during a consultation. It is intended to understand how feelings, such as gratitude, resilience, and forgiveness, and even spiritual conflicts affect a patient’s health. Patients’ degree of Spirituality and Religiosity can be evaluated in history or spiritual anamnesis because, for those who follow a religion or have strong sense of Spirituality, keeping it active during medical care brings numerous health benefits and improves the doctor-patient relationship (Avezum and Esporcatte, 2019).

There are different ways of including Spirituality in academic activities of medical schools, such as: elective courses; lectures; standardized patient interviews; chaplains’ follow-ups; reading scientific articles on Spirituality/Religiosity in clinical practice and related subjects; discussions in small groups; assembly of a practical setting for taking spiritual history (Fortin and Barnett, 2004; King et al., 2004).

The spiritual dimension stood out in the academic and scientific environment in the 1960s, when the first studies on Spirituality/Religiosity in clinical care were published (Puchalski, 2001b). In the academic context of Spirituality/Religiosity in Health, we should highlight the pioneering role of Christina Puchalski, who taught the first elective course in Spirituality and Health at the George Washington University School of Medicine in 1992 (Lucchetti et al., 2012b; Moreira-Almeida and Lucchetti, 2016).

Since then, several Faculties of Medicine have included courses and subjects on spirituality in their curriculum notes: in the United States more than 85%, in Canada 70% and in the United Kingdom between 31% and 59% (Neely and Minford, 2008; Lucchetti et al., 2012b; Moreira-Almeida and Lucchetti, 2016). In Brazil, 10.4% of the Faculties of Medicine have specific courses on spirituality and health and 40.5% included spirituality and health content in their disciplines (Lucchetti et al., 2012b).

Our research object that proposes the insertion of Spirituality/Religiosity in medical education in Brazil is anchored in the relevant approval data of this discipline in several countries, as mentioned earlier. In Brazil and in the world, there has been an increase in the number of Spirituality and Health research groups, events, funding, publications in high-impact journals and space in medical conferences, in addition to recommendations of the main international bodies for the inclusion of Spirituality in clinical care and health education, among other initiatives (Puchalski, 2001b; Modjarrad, 2004; Moreira-Almeida, 2007; Lucchetti and Granero, 2010; Lucchetti et al., 2012b).

We developed this study to verify the opinion of undergraduate medical students regarding the use of a patient’s Spirituality/Religiosity as a therapeutic resource in clinical practice and the inclusion of didactic-pedagogical actions in the medical curriculum. The sample consisted of 72% of the total population of EPM medical students whereas the samples by other authors, who also developed their studies with medical students distributed in the six undergraduate years on the theme of Spirituality/Religiosity in clinical practice, comprised a smaller portion of the population: 63% (Banin et al., 2013), 51.5% (Borges et al., 2013), and 60.3% (Lucchetti et al., 2014). The high rate of students’ support to our research shows that the theme on Spirituality/Religiosity is considered relevant in the Brazilian medical academic space. First step toward the insertion of changes is the availability and interest of addressing an unusual theme such as the insertion of Spirituality/Religiosity in medical courses. We present the distribution of research participants by religious affiliation: Catholic (29.7%), Evangelical Protestant (12.7%), Spiritist (11.7%), others religious denominations (7.3%), not affiliated/agnostic (26.2%), and not affiliated/atheist (12.4%) (Table 1).

In the academic scenario, as far as research is available, the *Duke Religious Index* (DUREL) (Koenig et al., 1997) presents high credibility, a relevant factor for us to include it in our research. According to Koenig and Büssing (2010), DUREL contemplates the different dimensions of religiosity related to health outcomes: organizational (OR) (Tables 2, 3), non-organizational (NOR) (Tables 4, 5) and intrinsic (IR) (Tables 6, 7). They also state that, although religious affiliation is an important fact, it tells us little about the student’s religiosity. The statistical test applied showed a significant difference between religious affiliation and students’ religiosity. The scores found were high of organizational religiosity (frequency to religious institution), non-organizational religiosity (individual religious experience) and intrinsic religiosity (centrality of religion in life) among students with religious affiliation; low among agnostics; and non-existent among atheists (Table 8). We also found a statistically significant positive correlation between all DUREL issues among themselves (Table 9).

As for Organizational Religiosity, the percentage we found of 51.8% of student’s who attended religious institutions “once a year and never” (Table 3) was much higher than the data found in studies that applied DUREL to medical students, such as the one by Lucchetti et al. (2014), who reported 36.6%. The participants of our research who have religious affiliation have a high frequency score at the religious temple (Table 8), and a statistically significant positive correlation between OR, NOR and IR, with simultaneous increase of variables (Table 9). We verified that Evangelicals were the ones with the highest religious frequency. Most Catholic, Spiritist and other denomination students attended religious institutions “a few times a year,” according to Uecker et al. (2007), the attendance to religious services occurred as a family habit that was lost upon starting the studies at the university.

The students’ Non-organizational Religiosity was measured by the time dedicated to individual religious activities. Students with religious affiliation have a high score of time dedicated to individual religious activities (Table 5), and a statistically significant positive correlation between NOR, OR and IR, with simultaneous increase in variables (Table 9). Our results showed that 22.1% of the students were dedicated to individual religious activities daily or more than once a day (Table 4), a considerably lower percentage than those found by Borges et al. (2013) and Lucchetti et al. (2014), respectively 38.8% and 32.7%. Uecker et al. (2007) reported that the low percentage of dedication to the practice of religious activities is due to the dazzle with the university and social life of students entering a world full of extra-religious activities. Among religious affiliations, 59.4% of Evangelicals are engaged in individual religious activities at least once a day, followed by 32.1% of Catholics. Dedication of at least once a week to these activities was reported by 45.4% of Spiritists and 42.5% of students from other religious affiliations (Table 5).

As for Intrinsic Religiosity, most Evangelicals had religious centrality varying from strong to very strong (82.6%). Already, 63% of Catholics, 62.5% Spiritists, and 67.5% of other religious denominations presented centrality ranging mostly from moderate to strong, suggesting that Religiosity occupies an important space in their lives (Table 7). Students with religious affiliation have a high score of centralities of religion in life (Table 8), and a statistically significant positive correlation between IR, OR and NOR, with simultaneous increase of variables (Table 9). Koenig et al. (2012) pointed out that there is an unequivocal relationship between good health indicators and the exercise of intrinsic Religiosity.

Discussing the results of DUREL, which showed us the religiosity of the research participants, we will go to the discussion of the third block. In it we will evaluate the degree of agreement of students to questions formulated, based on scientific literature, which see Spirituality/Religiosity within a clinical context. We will present questions numbers 12 to 18, discussing each of them.

From all study participants, 79.3% responded favorably to the proposition 12: “A physician should ask about spiritual questions that directly or indirectly influence a patient’s health positively” (Table 10). There was a statistically significant negative correlation between the year of graduation in which the student is and this question. As the student approaches his/her graduation, the agreement with the assertive one increases (Table 13). We also found a statistically significant positive correlation between this question and Intrinsic Religiosity. The higher the IR score, the higher the students’ level of agreement with the assertive (Table 13). Other studies have observed that most physicians recognize the importance and value of patients’ spiritual beliefs in their health and feel that they need to know such beliefs (Anandarajah and Hight, 2001; Chibnall and Brooks, 2001; Monroe et al., 2003; Puchalski, 2006; Lucchetti et al., 2010; Koenig, 2012a,b; Saad and De Medeiros, 2012; Puchalski et al., 2020). The same positive impact was observed in medical students in the samples by Banin et al. (2013) and Lucchetti et al. (2013b). Ellis et al. (1999) showed that most physicians (more than 90%) recognize that spiritual factors are an important health component and most of them (70% to 82%) state that this can influence a patient’s health. Moreover, the authors also reported that 85% of physicians said they should be aware of patients’ religious/spiritual beliefs, and 89% felt entitled to ask about such beliefs. These data corroborate our research object regarding the insertion of Spirituality/Religiosity in clinical practice.

In the proposition 13: “Regardless of their religious belief, a physician should respect a patient’s belief,” the degree of agreement was 98.6%. Three students opted for the “indifferent” alternative: a 1st-year atheist; a 3rd-year catholic; and a 6th-year agnostic. Four students marked “disagree”: two 2nd-year, agnostic; one of the third-year, atheist; and a fifth-year catholic. A single third-year student, a catholic, marked “I strongly disagree” (Table 10). We found a statistically significant positive correlation between this question and Intrinsic Religiosity. The higher the IR score, the higher the students’ degree of agreement with this statement (Table 13). Kørup et al. (2021) state that a physician should provide professional care, respecting and validating a patient’s beliefs, even if they disagree. The results by Barnett and Fortin (2006), in a study conducted with resident physicians and medical students, showed that a physician’s spiritual/religious beliefs could affect their ability to communicate and care for patients. They concluded that the strongest indicator for a physician to address spiritual needs or not is linked to that physician’s degree of Religiosity or Spirituality and not to a patient’s health condition. Chibnall and Brooks (2001) developed important work on the role of physicians’ religious beliefs in clinical practice and presented several suggestions to reduce physicians’ discomfort with spiritual anamnesis. Steinhauser et al. (2000) emphasized that detecting when the personal values of health professionals themselves based on a theistic or atheistic worldview-impact patient care is a daily challenge in clinical practice.

In the proposition 14: “A physician’s concern with a patient’s spiritual dimension demonstrates empathy and improves doctor-patient relationship,” we found an agreement of 92.5% (Table 10). There was greater agreement with this proposition among students who have religious affiliation, and among agnostics (Table 12). We confirmed a statistically significant positive correlation between organizational religiosity and this proposition. The higher the OR, the higher the level of agreement of the students (Table 13). We also found a statistically significant positive correlation between this question and Intrinsic Religiosity. The higher the IR score, the higher the students’ degree of agreement with this statement (Table 13). Some studies, such as the one by Peres et al. (2020), showed that many students agreed that patients should have their beliefs addressed and validated and that those beliefs could have an important impact, not only on the prognosis and outcome of the treatment, but also on the doctor-patient relationship. For D’Alessandro et al. (2020), the respect for a patient’s spiritual dimension allows a physician to better understand how a patient experiences the process of illness and to establish a deeper relationship with them. Frankl (1984) tells us that Spirituality can be a source of strength or a source of deep existential anguish. The author states that when, through the spiritual dimension, a physician establishes a connection with a patient, he/she achieves positive results such as those documented in several studies on the doctor-patient relationship. D’Alessandro et al. (2020) claim that patients may develop spiritual distress as the disease progresses. The authors stated that a patient often needs someone who is indeed present, willing to listen to their pains and anxieties to help them find answers, transcend, and reframe suffering. They concluded that it is very important for a doctor to be attentive, to listen empathically, and to establish a relationship of trust so that a patient can express their deepest anxieties.

The proposition 15: “A physician should consider a patient’s emotional, sociocultural, and spiritual needs as well as their physical needs in the clinical practice” received 97.1% agreement (Table 10). We confirmed a statistically significant positive correlation between this proposition and organizational religiosity. The higher the OR, the higher the level of agreement of the students (Table 13). We also found a statistically significant positive correlation between this question and Intrinsic Religiosity. The higher the IR score, the higher the students’ degree of agreement with this assertion (Table 13). The fact that medical students know the definition of Health recommended by WHO as “a state of complete physical, mental and social well-being and not only the absence of disease or illness” (World Health Organization [WHO], 1946) help them understand and agree with the above assertion. Due to the lack of consensus in the definition of Spirituality, the spiritual dimension was not included in the definition of Health (World Health Organization [WHO], 1999), but the WHO included it as a domain in its Quality-of-Life Instrument (WHOQOL-SRPB, 2002), emphasizing its importance.

The proposition 16: “Care for the spiritual dimension is part of a patient’s comprehensive and humanized care” obtained an agreement of 83.7% (Table 11). A significant difference was confirmed between Religious Affiliation and this proposition. There was greater agreement among students who have religious affiliation, and among agnostics (Table 12). We found a statistically significant positive correlation between this proposition and DUREL, the higher the DUREL scores (OR, NOR and IR), the higher the degree of agreement of the students to this question (Table 13). We also confirmed statistically significant positive correlations between the questions of the clinical context from 12 to 16 among themselves, which shows that the increase in the score of the variables is simultaneous (Table 14). In the Manual of Palliative Care, D’Alessandro et al. (2020) state that the recognition of spiritual needs is an essential part of patient-centered medicine. For many authors, an awareness of the importance of the spiritual dimension while treating the patient indicates the resurgence of a medical practice in which a human being should be valued in all their complexity (Peres et al., 2007; Lucchetti et al., 2013b; Reginato et al., 2016; Damiano et al., 2017) as well as the inclusion of spiritual anamnesis foreshadow the advent of a more humanized medicine (Reginato et al., 2016). Moreover, most physicians recognize in the relevance of this practice and a considerable number of patients yearn for an approach that includes their spiritual needs (Ellis et al., 1999; McCord et al., 2004; Puchalski, 2006; D’Souza, 2007; Saad et al., 2015; Reginato et al., 2016).

The proposition 17: “Denying his/her own emotions and distancing from a patient’s suffering is a defense strategy used by a physician” obtained agreement of 65.6% (Table 11). There was a statistically significant negative correlation between the year of graduation in which the student is and this proposition. As the student approaches his/her graduation, the agreement with the assertive one increases (Table 13). Some physicians, believing to be protecting themselves from suffering, create a barrier in their feelings and avoid seeing in the patient the fragility of the human being. It’s a way they find not to look at their own mortality (Marta et al., 2009).

The proposition 18: “A good doctor should distance himself/herself from a patient’s suffering” obtained 90.4% disagreement (Table 11). A statistically significant negative correlation was confirmed between organizational and intrinsic religiosities with this issue. The higher the organizational and intrinsic religiosities of the students, the lower the agreement with proposition 18 (Table 13). There were statistically significant negative correlations between propositions 14, 15, and 16 and this question. The higher the students agree with the related propositions, the lower the agreement with the assertive 18 (Table 14). As Kübler-Ross (2012) and Arantes (2016) advise us, a good doctor must learn to deal with empathy to be able to approach a patient without incorporating their pain. It is not an easy exercise, so they recommend the use of compassion, which consists of respect, care, mindfulness, and closeness, without the damage that can be caused by empathy.

The last question of our data collection instrument turned to the academic context. The statement 19: “The proposition of didactic-pedagogical actions related to a patient’s spiritual dimension in medical education is relevant” obtained 61.7% agreement. Only 11% of the students opposed these actions and 27.3% were indifferent (Table 11). There was greater agreement with this proposition among students who have religious affiliation, and lower among agnostics (Table 12). We found a statistically significant positive correlation between this proposition and DUREL, the higher the DUREL scores (OR, NOR and IR), the higher the degree of agreement of the students to this question (Table 13). We confirmed statistically significant positive correlations between the questions of the clinical context from 12 to 16 with proposition 19 of the academic context. The greater the students agree with the related propositions, the greater the agreement with this proposition (Table 14). There were statistically significant negative correlations between proposition 18 and this question. The higher the students agree with the related propositions, the lower the agreement with this assertive (Table 14). Corroborating our findings, Lucchetti et al. (2013b) found in their results that 61.6% of the students considered that a physician should be prepared to deal with spiritual issues related to their patients’ health and 62.6% were in favor of including this content in the medical curriculum. In the result obtained by Mariotti et al. (2011), although more than 72% of the medical professors investigated agreed that faith or Spirituality could positively influence their patients’ treatment, only 50% attributed importance to students’ preparation for this approach during the medical program.

During the research, we scored some limitations in our study. Because it is a cross-sectional survey, the students were not followed up during the academic training to know if their opinion about the propositions related to the Spirituality/Religiosity of the patient in clinical practice would change over time. We used a standardized questionnaire developed for this study, which can be influenced by social desirability, moderating the students’ responses, due to the social acceptability factor because they are inserted in a medical course. We included in our data collection instrument DUREL, which was designed to measure religiosity in Western religions and may be less accurate in its assessment of religiosity in Eastern religious traditions.

After these reflections, we will make some considerations regarding the conclusions of the research:

(1) Medical students attach significance to patient’s spiritual dimension in clinical practice: 79.3% are favorable to a physician ask a patient about spiritual issues; 98.6% agree that, regardless of their religious belief, a physician should respect a patient’s belief; 92.5% acknowledge that a physician’s concern with a patient’s spiritual dimension demonstrates empathy and improves doctor-patient relationship; 97.1% understand that a physician should consider a patient’s emotional, sociocultural, and spiritual needs as well as their physical needs in the clinical practice. Although 12.4% of the students declared themselves atheists, this did not interfere in the high percentage of agreement with the questions related to the importance of spiritual anamnesis. Many professionals still stand to the conviction that medicine should remain secular and unrelated to Spirituality/Religiosity as this subject is considered coercive by some patients (Lucchetti et al., 2012b). Our research proved that the majority of EPM medical students attach importance to the spiritual dimension of the patient in clinical practice. Studies corroborate our results by proving that the mere fact that a physician is concerned about a patient’s spiritual aspect can improve doctor-patient relationship and, consequently, the impact of performed medical interventions (Chibnall and Brooks, 2001; Peres et al., 2007; Berg et al., 2013; Lucchetti et al., 2013b; Reginato et al., 2016; Damiano et al., 2017).
(2) Students consider that caring for the patient’s spiritual dimension is part of the comprehensive and humanized patient care: 83.7% agree that caring for the patient’s spiritual dimension is part of the comprehensive and humanized patient care. Care for the spiritual dimension is the care of the “Spirituality/Religiosity of the patient” and should be used as a therapeutic resource in clinical practice through the taking of spiritual history (spiritual anamnesis) of the patient. A comprehensive and humanized patient care is aligned with the concept of total pain, proposed by Cicely Saunders, in 1967, which emphasizes the importance of interpreting the painful phenomenon not only in its physical dimension, but also in its emotional, social, and spiritual dimensions, affecting the genesis and expression of the painful complaint (Kübler-Ross, 2012; Arantes, 2016; D’Alessandro et al., 2020).
(3) The physician doesn’t should distance himself/herself from a patient’s suffering: 65.6% accept that the denial of their own emotions and the distancing from the patient’s suffering is a defense strategy used by a physician; 90.4% disagree that a good doctor should distance themselves from a patient’s suffering. According to Castelhano and Wahba (2019), the great challenge for most physicians is to recognize that it uses crystallized defensive attitudes that lead to an affective distancing from a patient. Associated with altruism, compassion is a fundamental tool for the exercise of medicine centered on a patient’s integral care that implies the recognition and care of others’ suffering, without sharing pain and without failing to look at oneself. All resources should be put into practice to alleviate a patient’s suffering, but this should not occur in the detriment of self-care and self-preservation (Puchalski, 2001a,^2006^; Kübler-Ross, 2012; Arantes, 2016; Reginato et al., 2016; D’Alessandro et al., 2020).
(4) Students are in favor of proposing didactic-pedagogical actions in medical education related to a patient’s spiritual dimension: 61.7% of the study participants considered these actions relevant and only 11% were contrary to them. Despite the scarcity of validated educational models to incorporate Spirituality courses in medical education and the lack of a national guideline on what should be included in the curriculum for this area, we should consider its importance and insertion as a discipline in medical schools in Brazil (Lucchetti et al., 2012b).

Our research object that proposes the insertion of the discipline of “Spirituality/Religiosity in clinical practice” in medical training in Brazil was anchored in relevant approval data in several countries. We verified the feasibility of incorporating Spirituality/Religiosity in clinical practice.

## Data Availability Statement

The raw data supporting the conclusions of this article will be made available by the authors, without undue reservation.

## Ethics Statement

The studies involving human participants were reviewed and approved by Research Ethics Committee/Federal University of São Paulo. The patients/participants provided their written informed consent to participate in this study.

## Author Contributions

This work was systematized by SB-A supported by her co-advisor LA and her advisor RT. All authors listed have made a substantial, direct, and intellectual contribution to the work, and approved it for publication.

## Conflict of Interest

The authors declare that the research was conducted in the absence of any commercial or financial relationships that could be construed as a potential conflict of interest.

## Publisher’s Note

All claims expressed in this article are solely those of the authors and do not necessarily represent those of their affiliated organizations, or those of the publisher, the editors and the reviewers. Any product that may be evaluated in this article, or claim that may be made by its manufacturer, is not guaranteed or endorsed by the publisher.

## Footnotes

1 Available in: https://www.who.int/about/governance/constitution. Accessed: 28 Oct. 2021.

2 The taking of the patient’s spiritual history, also called spiritual anamnesis, seeks to raise his belief: it brings comfort or generates stress; gives purpose and meaning to his life; helps in coping with diseases; has some restriction that affects treatment; enables you to be part of a spiritual community that supports you. For non-religious patients, one should ask what gives meaning and purpose to their life, how they live with the disease and what impact their cultural beliefs can cause on their treatment (Koenig, 2012a).

3 The anamnesis (from greek, *ana* = bring again; *mnesis* = memory) involves the core of the doctor-patient relationship and guides the diagnostic and therapeutic plan. In summary, it is an interview that aims to bring back to the patient’s mind all the facts related to the disease, signs and symptoms, through which it will be possible to trace a diagnostic hypothesis and establish an appropriate treatment approach (Santos et al., 2003; Soares et al., 2014).

4 We emphasize that there was no promise of bonuses to the students participating in the survey.
