# Corrigendum: Spirituality/Religiosity as a Therapeutic Resource in Clinical Practice: Conception of Undergraduate Medical Students of the Paulista School of Medicine (*Escola Paulista de Medicina*) - Federal University of São Paulo (*Universidade Federal de São Paulo*)

**DOI:** 10.3389/fpsyg.2022.886507

**Published:** 2022-03-29

**Authors:** Silvia Borragini-Abuchaim, Luis Garcia Alonso, Rita Lino Tarcia

**Affiliations:** ^1^Center for the Development of Higher Education in Health (CEDESS), Paulista School of Medicine, Federal University of São Paulo, São Paulo, Brazil; ^2^Department of Morphology and Genetics, Paulista School of Medicine, Federal University of São Paulo, São Paulo, Brazil

**Keywords:** spirituality, religiosity, medical education, clinical practice, undergraduate medical students

In the original article, there was an error in the text as published. The term “ciência da religião” was wrongly translated as “science of religion” and “cientista da religião” as “scientist of religion.” The correct terms for English native speakers would be “study of religion” and “scholar of religion,” respectively.

A correction has been made to *Discussion, Paragraphs 4, 5, and 6*. The corrected paragraphs are shown below.

Stern (2017) states that the differentiation between religion, religiosity and spirituality is very difficult to trace and points out several problems in the WHO definitions from the perspective of the scholar of religion. Initially, the definitions of religion and religiosity are based exclusively on the model of Abrahamic religions. By using the term “Spirituality,” which would not necessarily relate to any religion, the WHO supports physicians to act in this field without hurting their codes of ethics but does not identify elements that can be only “spiritual,” without being “religious.”

The scientific literature presents a wide variety of works that deal with the inclusion of the Discipline of Spirituality and Religiosity in the medical curriculum. However, in pedagogical projects there is no specification of the most indicated professional category to coordinate this course and teach the classes. Stern (2018) believes that it is possible, with the application of concepts from the study of religion, to build professional bridges between study of religion and health professionals. By the specific training in the theme and acquired skills, the scholar of religion has the most appropriate profile to train medical students in Spirituality/Religiosity.

We agree that academic training does not empower physicians, future teachers, to use the spiritual/religious context in clinical practice, therefore, they will not be able to transmit to their students a knowledge they do not have. It is necessary that a multidisciplinary team act, led by a scholar of religion, who can teach students the bases of religious traditions, so diverse in Brazil, so that when they receive, for example, a patient who is a Jehovah's Witness know that they will not be able to perform blood transfusion without authorization. In the prescription of medications, other religions have restrictions on substances, days of the week, schedules. The scholar of religion will be able to guide students on how to detect spiritual suffering and approach the patient about wanting to talk to the chaplain or, if not, with the religious leader of his religious belief. The doctor can work together with the scholar of religion to explain to the students how to proceed with the taking of spiritual history. Validated simple instruments, as FICA [F (Faith/belief)/I (Importance or influence)/C (Community)/A (Action in treatment)] (Puchalski and Romer, 2000) and HOPE [H (Sources of Hope)/O (Organized Religion)/P (Personal spirituality and practice)/E (Effects on medical treatment and terminal matters)] (Anandarajah and Hight, 2001), for example, can detect, in a matter of minutes, if the patient needs spiritual care, not for the doctor to treat him, but to refer him to the qualified professional.

The authors apologize for these errors and state that they do not change the scientific conclusions of the article in any way. The original article has been updated.

## Publisher's Note

All claims expressed in this article are solely those of the authors and do not necessarily represent those of their affiliated organizations, or those of the publisher, the editors and the reviewers. Any product that may be evaluated in this article, or claim that may be made by its manufacturer, is not guaranteed or endorsed by the publisher.
